# Associations between Heart Rate Variability Parameters and Hemodynamic Profiles in Patients with Primary Arterial Hypertension, Including Antihypertensive Treatment Effects

**DOI:** 10.3390/jcm11133767

**Published:** 2022-06-29

**Authors:** Małgorzata Maciorowska, Paweł Krzesiński, Robert Wierzbowski, Beata Uziębło-Życzkowska, Grzegorz Gielerak

**Affiliations:** Department of Cardiology and Internal Diseases, Military Institute of Medicine, ul. Szaserów 128, 04-141 Warsaw, Poland; pkrzesinski@wim.mil.pl (P.K.); rwierzbowski@wim.mil.pl (R.W.); buzieblo-zyczkowska@wim.mil.pl (B.U.-Ż.); ggielerak@wim.mil.pl (G.G.)

**Keywords:** autonomic nervous system dysfunction, heart rate variability, hypertension, hemodynamic profile

## Abstract

Background: Autonomic nervous system (ANS) dysfunction is an important factor in the development and progression of arterial hypertension (AH) and may produce adverse hemodynamic sequelae. ANS function can be evaluated by analyzing heart rate variability (HRV). The purpose of this study was to assess the possible correlation between HRV and the hemodynamic profile of AH patients, including antihypertensive treatment effects after 12 months. Methods: The study was conducted on 144 patients with uncomplicated AH. The hemodynamic profile was assessed via echocardiography and impedance cardiography (ICG). The analyzed HRV parameters included SDNN, rMSSD, pNN50, low frequency (LF, 0.05–0.15 Hz), high frequency (HF, 0.15–0.4 Hz), total power (TP, the variance of all NN intervals), and the day, night, and 24-h low-to-high frequency ratios (LF/HF). Results: Analysis showed various correlations of HRV parameters both with arterial blood pressure and with the hemodynamic profile assessed via echocardiography and ICG. The HRV parameters of increased ANS activity showed a correlation with improved left ventricle function (LV) and lower LV afterload. Conclusions: Effective antihypertensive treatment demonstrated beneficial effects on both the ANS balance and the hemodynamic profile.

## 1. Introduction

Arterial hypertension (AH) develops in large part due to interactions between complex vascular, neurogenic, hormonal, renal, and metabolic regulatory mechanisms. Often, there is a functional imbalance of the autonomic nervous system (ANS) with sympathetic dominance. This chronic, increased sympathetic activity may lead to adverse hemodynamic effects, including arterial and arteriolar vasoconstriction, increased systemic vascular resistance, and cardiac cycle disturbances [1,2].

One of the methods used in assessing ANS function is heart rate variability (HRV) analysis, which may be conducted based on electrocardiographic tracings from 24-h Holter monitoring [3,4,5,6,7]. Hemodynamic analysis, which can be achieved via echocardiography or other noninvasive techniques, e.g., impedance cardiography (ICG) [8,9,10,11,12,13,14,15], is of significant informative value, as it can detect cardiovascular dysfunction already at a subclinical stage [8,9,14,15,16], and of therapeutic value, as it helps adjust antihypertensive treatment to the leading underlying mechanism of hypertension [11,12].

Analysis of ANS correlations with the cardiovascular system function is one of the key aspects of the research on AH pathophysiology [2,17,18] and may have important clinical implications. Therefore, the purpose of this study was to assess the correlations between HRV and both the arterial blood pressure (BP) and the hemodynamic parameters assessed via echocardiography and ICG, including the effects of 12-month antihypertensive treatment.

## 2. Methods

### 2.1. Study Population

The present study is a secondary analysis of the data collected as part of the FINEPATH study (ClinicalTrials.gov NCT01996085). This was a prospective, randomized controlled trial; its primary objective was to assess a novel method of selecting medical treatment for patients with AH [12]. The design of the present study had been approved by the Bioethics Committee at the Military Institute of Medicine (approval No. 21/WIM/2011), and each patient provided written informed consent. The study population was a group of 144 patients with AH, defined according to the European Society of Cardiology (ESC) guidelines [19] as BP values of ≥140/90 mmHg for at least 3 months prior to study inclusion. Recruitment was conducted over the period from the year 2011 to 2014.

Study inclusion criteria were: documented secondary AH, chronic kidney disease (an estimated glomerular filtration rate of <60 mL/min/1.73 m^2^ calculated with the Modification of Diet in Renal Disease (MDRD) study equation [20]), severe comorbidities (systolic heart failure, cardiomyopathies, significant valvular heart disease, chronic obstructive pulmonary disease, preexisting diabetes mellitus, polyneuropathy, peripheral vascular disease, significant arrhythmias), non-sinus rhythm (including permanent pacing), age above 18 and under 75 years, body mass index (BMI) >40 kg/m^2^, and mental conditions precluding full patient cooperation. The patients already receiving antihypertensive treatment were advised to discontinue it at least 7 days prior to the initial assessment, after which their further medical treatment was prescribed, with the use of the following drug classes in monotherapy or combination therapy: beta-blockers, angiotensin-converting enzyme inhibitors (ACE-I), angiotensin receptor blockers (ARB), diuretics, and calcium channel blockers (CCB) [12]. Each patient was evaluated twice—at study inclusion and after 12 months of antihypertensive treatment.

### 2.2. 24-H Ambulatory Blood Pressure Monitoring (ABPM)

The presence of AH was confirmed via 24-h ABPM with the use of a Spacelabs 90207 device (Medical Inc., Snoqualmie, WA, USA). The automatic BP measurements were conducted in 10-min intervals during the day (6:00 a.m.–10:00 p.m.) and in 30-min intervals during the night (10:00 p.m.–6:00 a.m.). The cutoff points for AH diagnosis were adopted in accordance with the guidelines in force at the time [19].

### 2.3. 24-H Electrocardiography (Holter-ECG)

All patients underwent 24-h electrocardiographic recording (Holter) with the use of 3-channel digital LifeCard CF devices (Del Mar Reynolds Medical—Spacelabs Healthcare, WA, USA). Routine assessments for arrhythmias and the minimum, mean, and maximum heart rate (HR) were expanded to include HRV. Data analysis, including the frequency and time domains of HRV, was conducted with an Impresario Symphony Holter Analyzer (Del Mar Reynolds Medical, Spacelabs Healthcare Ltd., Hertford, UK). Electrocardiographic signal preprocessing included manual correction of imperfectly classified beats, artifact elimination, and arrhythmia analysis. Only the R–R intervals between normal QRS complexes were analyzed, whereas the R–R intervals immediately before and after an extrasystole were disregarded. Recordings containing >300 extrasystoles and artifacts were excluded from further analysis.

### 2.4. Heart Rate Variability (HRV) Analysis

The time domain was analyzed automatically and included the standard deviation of the average of NN (normal-to-normal R-R interval) intervals (SDNN), the square root of the mean of the sum of the squares of differences between adjacent NN intervals (rMSSD), and the proportion of pairs of successive NN intervals that differ by more than 50 ms (pNN50). All these parameters are related to autonomic balance and represent mainly its parasympathetic arm [3,4,5,6,7]. The full analysis included all these parameters for 24 h period (*parameter*_24 h), day-time (*parameter*_day), and night-time (*parameter*_night).

The frequency domain was analyzed with a fast Fourier transform (FFT), following sampling with cubic-spline interpolation and Blackman–Harris window function. The spectrum of HRV was divided into low frequency (LF, 0.05–0.15 Hz), high frequency (HF, 0.15–0.4 Hz) oscillations, the LF/HF ratio, and total power (TP). A frequency analysis was performed for each hour out of the 24-h period. Subsequently, the mean values for the day and night and the day/night ratio were calculated. The HF or so-called respiratory band (0.15–0.40 Hz) is influenced by breathing and represents the cardiac vagal control, while the LF band is influenced by both parasympathetic and sympathetic nervous systems. The low LF/HF ratio reflects parasympathetic dominance, and in 24 h recordings, it is assumed that its low value is a product of parasympathetic dominance [3,4,5,6,7].

### 2.5. Echocardiography

Resting two-dimensional echocardiography was conducted with standard parasternal and apical views (a VIVID S6 device, GE Medical System, Wauwatosa, WI, USA). Global assessment of systolic left ventricular (LV) function was conducted by measuring the left ventricular ejection fraction (LVEF) with the use of a modified biplane Simpson’s method and global longitudinal strain (GLS). Diastolic function was assessed with pulsed-wave Doppler and tissue Doppler techniques, assessing the following variables: E/A wave ratio (the mitral flow velocity in an apical four-chamber view, pulsed wave Doppler at the mitral leaflet tips)—where the E wave reflects the early diastolic pressure gradient between the left atrium and the left ventricle (LV), which is a derivative of LV diastolic function and left atrial pressure, whereas the A wave reflects the end-diastolic pressure gradient between the left atrium and LV, which correlates with left atrial systolic function; e’ wave velocity measured at the septal side of the mitral annulus (a four-chamber view, with the selected pulsed-wave Doppler registration gate located 5–10 mm above the mitral annulus near the basal segments of the interventricular septum); and the E/e’ ratio.

### 2.6. Impedance Cardiography (ICG)

ICG measurements were conducted with the use of a Niccomo^TM^ device (Medis, Ilmenau, Germany) in a supine position after a 10-min rest. The analysis included the calculated mean values of LV function variables, such as the stroke index (SI), cardiac index (CI), velocity index (VI), acceleration index (ACI), and Heather index (HI). Concurrent systolic blood pressure (SBP) and diastolic blood pressure (DBP) measurements conducted in 2-min intervals with an arm cuff also allowed for calculating such hemodynamic parameters as the systemic vascular resistance index (SVRI) and total artery compliance (TAC).

## 3. Statistical Analysis

Statistical analysis was conducted with Statistica 12.0 (StatSoft Inc., Tulsa, OK, USA). The distribution and normality of the data were assessed visually and with the Kolmogorov–Smirnov test. Continuous variables have been presented as means ± standard deviation (SD), whereas nominal categorical variables have been presented as numbers and proportions. The change from baseline in the evaluated variables at month 12 of treatment was calculated with the formula: delta_X = [12-month X value] − [baseline X value]. The Wilcoxon signed-rang test was used to assess the effect of treatment. The correlation between variables was assessed with Pearson’s and Spearman’s correlation. A *p*-value of < 0.05 was considered statistically significant.

## 4. Results

### 4.1. Baseline Characteristics of the Study Population

**Figure 1:**
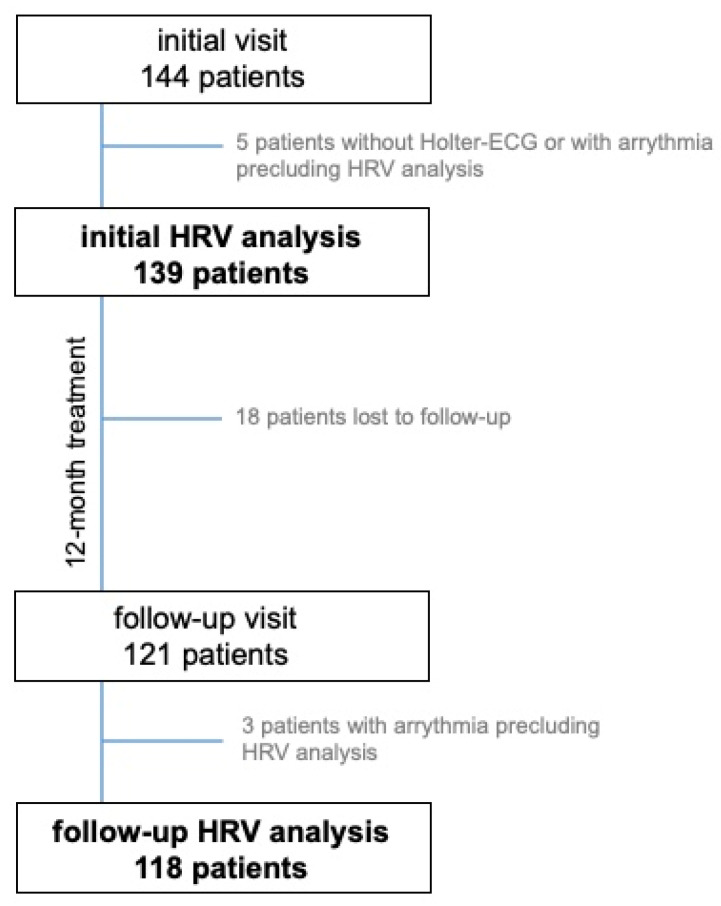
Study group flowchart. HRV—heart rate variability.

An HRV analysis based on the 24-h Holter ECG from the initial visit was possible in 139 out of 144 patients included in the FINEPATH study. The effects of treatment were assessed after 12 months in 118 patients who underwent Holter ECG at the follow-up visit. Eighteen patients were lost to follow-up, and in three out of those who underwent follow-up Holter ECG, the analysis was impossible Figure 1.

Males constituted 69% of the study group. The mean age was 45 ± 11 years, mean BP was 141 ± 13/90 ± 9 mmHg, and mean HR was 73 ± 11/min. Most of the patients (81.0%) were individuals with mild (grade 1) AH. Slightly more than half of the individuals from the study group met the diagnostic criteria of metabolic syndrome [21]. Only 20.7% of study participants received antihypertensive medications prior to being included in the study, and these medications were discontinued at least 7 days prior to screening.

Study group characteristics have been presented in Table 1. The results of echocardiographic and HRV assessments have been summarized in Supplementary Materials Tables S1 and S2.

After 12 months of antihypertensive treatment, whose profile has been presented in Figure S1, we observed a significant BP reduction to 121/77 mmHg on average (*p* < 0.001). A similar effect was observed in terms of HR (68/min; *p* < 0.001) Figure S2. The mean body weight in the study group did not change over the 12-month period (87 ± 16 kg prior to vs. 87 ± 15 kg after treatment; *p* = 0.84).

### 4.2. The Association between HRV Parameters and Blood Pressure

Baseline DBP values showed a negative correlation with HRV indices of vagal control: SDNN, rMSSD, and pNN50. After the 12-month treatment period, we observed a negative correlation between changes in SBP/DBP and changes in SDNN and rMSSD—Table 2. No significant association between frequency-domain HRV parameters and BP was noted. Detailed results are presented in Table S3.

### 4.3. The Association between HRV Parameters and Echocardiographic Indices of LV Systolic and Diastolic Function

Some baseline HRV indices of parasympathetic function (SDNN, rMSSD, pNN50) and TP correlated positively with E/A wave ratio and e’—Table 3. For the same parameters, the only significant correlations between changes after 12-month treatment were noted for rMSSD and pNN50 with LVEF and E/A. Additionally, some positive correlations between GLS and some day-time HRV indices of vagal tone were noted for changes after treatment—detailed results are presented in Table S4.

### 4.4. The Association between HRV Variables and Impedance Cardiography Parameters

The baseline HRV indices of parasympathetic function (SDNN, rMSSD, pNN50, HF, LF/HF) and TP were positively correlated with most hemodynamic indices of left ventricular performance (SI, VI, ACI, HI), while for the LF and LF/HF ratio, the correlations were negative—Table 4. On the contrary, HR was negatively related to the level of cardiac vagal control. Only incidental correlations were present for HRV and measures of vascular compliance (SVRI, TAC).

Similar relations were observed in the analysis of changes—the improvement in left ventricular performance was related to the shift of the autonomic balance towards vagal control. Some correlations between increased parasympathetic function (SDNN, rMSSD, pNN50) and better vascular function (SVRI, TAC) also appeared.

## 5. Discussion

Our comprehensive assessment of the hemodynamic profile revealed various associations between the evaluated HRV variables and the hemodynamic profile in relatively young patients with AH without clinically significant comorbidities. Although the power of these correlations was low, they were consistent, and their combined interpretation indicates that mild LV dysfunction and increased LV afterload are closely associated with ANS imbalance already at the subclinical stage. Our results also suggest that the improvement in the hemodynamic profile is related to the shift in autonomic balance towards its parasympathetic arm.

The study included young and middle-aged patients with mostly mild AH and no significant comorbidities. Individuals with LV hypertrophy and diastolic dysfunction constituted a small proportion of the study participants. Slightly over half of the study population were individuals who met the diagnostic criteria for metabolic syndrome [21]. Only one in five study patients had received antihypertensive medications prior to study inclusion, and these were discontinued at least 7 days prior to HRV and hemodynamic profile assessments. All this leads us to believe that the adopted methodology ensured a reliable, relatively unconfounded assessment of the effects of AH on the combined function of the autonomic and cardiovascular systems.

Baseline assessments revealed a correlation between time-domain HRV variables and DBP, whereas the post-treatment follow-up assessments revealed the changes in time-domain HRV variables to be negatively correlated with the changes in both SBP and DBP. This indicates an association between elevated BP values and increased ANS activity and a beneficial and interrelated effect of antihypertensive treatment on both parameters. Our study results are consistent with those reported in the literature. In a Japanese observational study from 2014, Mori et al. [22] analyzed 5-min electrocardiographic recordings in 3458 individuals aged 40–74 years (51% of the male and 45.1% of the female patients had AH). A multivariate analysis showed a negative correlation between SDNN, rMSSD, LF, and HF on the one hand and the DBP value on the other; it also showed a positive correlation between those variables and the LF/HF ratio in both sexes. An increase in SDNN by one SD was associated with a drop in DBP by 1 mmHg in males and by 1.21 mmHg in females (after adjustment for age and other covariates). In women, SDNN, rMSSD, and HF also showed a negative correlation with SBP, whereas in men, there were no statistically significant correlations. Correlations between HRV and BP were not statistically significant in patients on antihypertensive treatment. The ARIC study showed an association between a higher BP and a considerably lower HRV in the entire study cohort. In addition, the correlations were stronger for DBP than for SBP, with greater differences observed in individuals without antihypertensive treatment [23]. After covariates and baseline BP levels were taken into account, the Framingham Heart Study revealed an association between a decrease in LF by 1 standard deviation and an increase in the mean SBP by 1.96 mmHg and in DBP by 0.83 mmHg in men, and in SBP by 0.7 mmHg and in DBP by 0.26 mmHg in women [24].

Our study group showed a correlation between HRV parameters (primarily in the time domain) and selected echocardiographic variables of diastolic LV function, i.e., the E/A wave ratio and e’ (however, there was no correlation with the E/e’ ratio). Changes in HRV variables also correlated with changes in the above echocardiographic variables after 12 months of treatment. Out of the measurements conducted at baseline, only one variable (TP) of spectral analysis of HRV correlated with systolic LV function assessed via LVEF. However, after 12 months of treatment, changes in LVEF correlated with changes in HRV parameters also in the time-domain analysis. At baseline, there was no correlation between HRV and GLS; however, follow-up data analysis indicated that changes in GLS correlated with changes in HRV variables in time-domain analysis (a positive correlation) and with the LF/HF ratio (a negative correlation). These results indicate an association between ANS dysfunction and subclinical myocardial dysfunction. There are few available original articles that focus on this topic. A study from an Italian center demonstrated that the LF ratio measured in patients in an upright position is higher in hypertensive patients with diastolic dysfunction than in those without (*p* < 0.05). LF measured at rest showed a significant correlation with the E/A wave ratio and, after tilt, with the E-wave deceleration time [25]. In patients with untreated hypertension, Tadic et al. demonstrated a correlation between the E/e’ ratio as well as the LV longitudinal, circumferential, and three-dimensional deformation on one hand and time-domain and spectral HRV variables on the other [26]. A multivariate regression analysis showed the diastolic function, longitudinal strain, and three-dimensional deformation each to be independently associated with HRV variables (SDNN, rMSSD, LF, HF, and TP). Moreover, in comparison with controls, patients with AH showed lower time-domain (SDNN, SDANN, pNN50, and rMSSD) and spectral variables (HF, LF, TP) except for the LF/HF ratio, which showed no differences between groups [26]. A study by Konrady et al. evaluated HRV in patients with various types of hypertension-associated LV remodeling. A head-up tilt test in normotensives revealed a considerable increase in the LF/HF ratio, which was largely due to lowered HF values. Patients with AH and normal LV geometry showed a three-fold increase in the LF/HF ratio, whereas individuals with either concentric or eccentric LV remodeling showed either no such reaction or even an inverse reaction to passive tilt. Correlation analysis revealed that increased left ventricular mass index (LVMI) and relative wall thickness (RWT) in patients with LV hypertrophy were associated with a reduction in HRV variables (r = −0.43 and r = −0.36, respectively; *p* < 0.001). Patients with impaired relaxation showed lower HF values (r = 0.4; *p* < 0.001) and a negative correlation between the E/A wave ratio and the LF/HF ratio. Moreover, those authors reported a negative correlation between HRV (TP) and both SBP (r = −0.51; *p* < 0.001) and DBP (r = −0.45; *p* < 0.001) [27].

Additional informative value is provided by the various correlations between HRV variables and the ICG-assessed hemodynamic parameters. HRV indicators of a shift in ANS balance towards sympathetic predominance showed a relationship with LV dysfunction (SI, ACI, VI, HR) and higher LV afterload (SVRI, TAC). These results suggest that already at an early stage of AH development before advanced complications become apparent, there is hemodynamic dysfunction of the cardiovascular system both associated with and, likely, induced by ANS dysfunction. This is consistent with the data available in the sparse literature on this topic [10,28]. A study by Abdelhammed et al. [28] showed that individuals with high normal BP values had a lower TAC index (TACI) in comparison with those with BP below 120/80 mmHg. Patients with AH showed considerably lower SI, CI, TACI, and TFC values, unlike SVRI values, which were higher [28]. Floras and Hara [29] demonstrated increased ANS activity (assessed via microneurography) and increased cardiac output in younger patients with AH (mean age 31 years, mean BP 151/95 mmHg) in comparison with the normotensive ones. In a study mentioned above, Aoka et al. [10], who evaluated 240 patients with untreated primary hypertension at baseline, reported high peripheral vascular resistance and a normal or low CI in 67% of patients and a high CI with normal SVRI in 16% of patients. Importantly, our study demonstrated that antihypertensive treatment has a beneficial effect on both HRV and the hemodynamic profile and that these phenomena are associated with each other.

## 6. Limitations

While interpreting the results of our study, it is important to consider the difficulties in comparing this study with earlier ones due to the differences between study protocols and the lengths of analyzed electrocardiographic recordings. Moreover, there have been few studies with a separate group of patients with uncomplicated AH, which corresponds to patient characteristics in the FINEPATH study. Nonetheless, we would like to emphasize that one strength of this study is the homogeneity of the study group and the lack of clinically significant comorbidities. This reduced a potentially confounding effect of comorbidities on ANS function; however, this also precludes the extrapolation of our results onto other patients with AH. We also made efforts to eliminate the effects of medical treatment used prior to the study in 21% of the study population; nevertheless, we cannot exclude the possibility of such effects persisting for longer than the set washout period of minimum 7 days prior to initial assessments. Moreover, the effect of different treatment regiments, potentially possible according to the previous reports [30,31,32], was not analyzed because of the small sample size for particular subgroups (Figure S1). We are aware that the power of the correlations was quite low, but the combined evaluation enabled rational scientific and clinical interpretation. The consistency of the results of the multiple tests suggests that the potential risk of type I error is low. Undoubtedly, further studies in larger groups could provide data for the more advanced multivariable analysis.

## 7. Conclusions

Patients with AH and no significant comorbidities showed: (1) an association between HRV and both arterial BP and the hemodynamic profile, both in terms of echocardiographic and ICG variables—indicators of increased sympathetic activity correlated with poorer systolic and diastolic LV function; (2) an association between a beneficial effect of 12-month antihypertensive treatment in the form of improved hemodynamic factors, with improved ANS function.

## Figures and Tables

**Table 1:** Baseline clinical data.

	Mean ± SD/n (%)
Age (years)	45.2 ± 10.5
Males	96 (69)
HR (1/min)	73.4 ± 10.7
SBP (mmHg)	141.2 ± 13.1
DBP (mmHg)	90.2 ± 9.3
MS IDF	81 (58.3)
Creatinine (mg/dL)	0.83 ± 0.16
eGFR (mL/min/1.73 m^2^)	99.9 ± 18.4
Glucose (mg/dL)	98.6 ± 11.2
HDL (mg/dL)	57.8 ± 18.5
LDL (mg/dL)	144.3 ± 34.6
TG (mg/dL)	155.2 ± 76.8
BMI (kg/m^2^)	29.1 ± 4.2

BMI—body mass index; DBP—diastolic blood pressure; eGFR—estimated glomerular filtration rate; HDL—high-density lipoprotein; HR—heart rate; LDL—low-density lipoprotein; MS IDF—metabolic syndrome in accordance with the International Diabetes Foundation criteria [21]; SBP—systolic blood pressure; SD—standard deviation; TG—triglycerides.

**Table 2:** Correlations of heart rate variability parameters with systolic and diastolic blood pressure for baseline absolute values and changes (delta) after 12-month treatment.

Baseline—R (Correlation Coefficient)		
HRV Parameters	SBP (mmHg)	DBP (mmHg)
SDNN_24 h (ms)	−0.06	−0.23 **
rMSSD_24 h (ms)	−0.07	−0.23 **
pNN50_24 h (%)	−0.06	−0.21 *
Change after treatment (delta)—R (correlation coefficient)		
	SBP (mmHg)	DBP (mmHg)
SDNN_24 h (ms)	−0.28 **	−0.31 ^#^
rMSSD_24 h (ms)	−0.21 *	−0.40 ^#^
pNN50_24 h (%)	−0.04	−0.10

Statistically significant correlations: * *p* < 0.05; ** *p* < 0.01; ^#^ *p* < 0.001. DBP—diastolic blood pressure; HRV—heart rate variability; pNN50—the proportion of pairs of successive NN intervals that differ by more than 50 ms; rMSSD—the square root of the mean of the sum of the squares of differences between adjacent NN intervals; SBP—systolic blood pressure; SDNN—standard deviation of the average of NN intervals.

**Table 3:** Correlations of heart rate variability parameters with echocardiographic parameters of LV systolic and diastolic function for baseline absolute values and changes (delta) after 12-month treatment.

Baseline—R (Correlation Coefficient)					
HRV Parameters	LVEF	E/A	e’	E/e’	GLS
SDNN_24 h (ms)	−0.01	0.36 ^#^	0.23 **	−0.03	−0.09
rMSSD_24 h (ms)	−0.05	0.41 ^#^	0.24 **	−0.04	−0.01
pNN50_24 h (%)	−0.07	0.44 ^#^	0.23 **	−0.01	−0.01
TP_day (ms^2^)	0.06	0.32 ^#^	0.26 **	−0.17	−0.09
TP_night (ms^2^)	−0.17 *	0.28 **	0.23 **	−0.06	0.03
Change after Treatment (Delta)—R (Correlation Coefficient)					
HRV Parameters	LVEF	E/A	e’	E/e’	GLS
SDNN_24 h (ms)	0.14	0.10	0.15	−0.09	0.06
rMSSD_24 h (ms)	0.25 **	0.34 ^#^	0.13	−0.03	0.19
pNN50_24 h (%)	0.22 *	0.38 ^#^	0.18	−0.03	0.16
TP_day (ms^2^)	0.09	0.07	0.09	−0.07	−0.04
TP_night (ms^2^)	0.14	0.17	0.10	−0.06	0.03

Statistically significant correlations: * *p* < 0.05; ** *p* < 0.01; ^#^ *p* < 0.001. delta—change after 12-month treatment; e’—mitral septal annulus early diastolic velocity; E/A—the ratio of the early (E) and late (A) mitral flow; E/e’—the ratio of early mitral flow (E) and mitral septal annulus early diastolic velocity (e’); GLS—global longitudinal strain; HF—high frequency; HRV—heart rate variability; LF—low frequency; LVEF—left ventricular ejection fraction; ns—not statistically significant; n.u.—normalized units; pNN50—the proportion of pairs of successive NN intervals that differ by more than 50 ms; rMSSD—the square root of the mean of the sum of the squares of differences between adjacent NN intervals; SDNN—standard deviation of the average of NN intervals; TP—total power.

**Table 4:** Correlations of heart rate variability parameters with impedance cardiography parameters for baseline absolute values and changes (delta) after 12-month treatment.

Baseline—R (Correlation Coefficient)								
HRV Parameters	SI	CI	HR	VI	ACI	HI	SVRI	TAC
SDNN_24 h (ms)	0.27 *	0.02	−0.35 ^#^	0.27 **	0.27 **	0.15	−0.15	0.17 *
rMSSD_24 h (ms)	0.28 ^#^	0.03	−0.42 ^#^	0.33 ^#^	0.32 ^#^	0.22 *	−0.13	0.13
pNN50_24 h (%)	0.30 ^#^	0.04	−0.46 ^#^	0.40 ^#^	0.39 ^#^	0.27 **	−0.14	0.14
LF/HF_day (-)	−0.18 *	−0.13	0.10	−0.17	−0.15	−0.29 ^#^	0.14	−0.04
LF_day (n.u.)	−0.17	−0.13	0.09	−0.15	−0.12	−0.30 ^#^	0.14	−0.01
HF_day (n.u.)	0.19 *	0.13	−0.12	0.19 *	0.17	0.31 ^#^	−0.14	0.03
TP_day (ms^2^)	0.12	−0.01	−0.21 *	0.21 *	0.22 *	0.08	−0.06	0.17
LF/HF_night (-)	−0.16	−0.03	0.28 ^#^	−0.24	−0.22 *	−0.28 ^#^	0.090	−0.00
LF_night (n.u.)	−0.11	−0.04	0.22 **	−0.19 *	−0.18 *	−0.27 **	0.10	0.04
HF_night (n.u.)	0.17	0.04	−0.30 ^#^	0.26 **	0.24 **	0.27 **	−0.08	0.03
TP_night (ms^2^)	0.15	0.12	−0.23 **	0.18 *	0.17 *	0.06	−0.05	0.18 *
Change after treatment (delta)—R (correlation coefficient)								
HRV parameters	SI	CI	HR	VI	ACI	HI	SVRI	TAC
SDNN_24 h (ms)	0.20 *	0.01	−0.19 *	0.18	0.12	0.02	−0.15	0.20 *
rMSSD_24 h (ms)	0.34 ^#^	−0.01	−0.32 ^#^	0.27 **	0.23 *	0.15	−0.21 *	0.22 *
pNN50_24 h (%)	0.47 ^#^	0.05	−0.43 ^#^	0.34 ^#^	0.31 ^#^	0.20 *	−0.26 **	0.27 **
LF/HF_day (-)	−0.06	−0.05	−0.01	0.000	−0.03	−0.22	0.18	−0.02
LF/HF_night (-)	−0.26 **	−0.08	0.22 *	−0.19 *	−0.21 *	−0.19 *	0.11	−0.15
LF_day (n.u.)	0.01	−0.05	−0.09	0.03	0.01	−0.18	−0.15	0.01
LF_night (n.u.)	0.21 *	−0.09	0.15	−0.16	−0.15	−0.18	0.11	−0.12
HF_day (n.u.)	0.02	−0.01	−0.03	0.004	0.04	0.16	−0.14	0.11
HF_night (n.u.)	0.28 **	0.08	−0.23 *	0.23 *	0.21 *	0.19 *	−0.10	0.13
TP_day (ms^2^)	0.03	−0.11	−0.13	−0.01	0.01	−0.01	−0.003	0.07
TP_night (ms^2^)	0.17	0.02	−0.14	0.13	0.13	0.19 *	−0.05	0.001

Statistically significant correlations: * *p* < 0.05; ** *p* < 0.01; ^#^ *p* < 0.001. ACI—acceleration index; CI—cardiac index; delta—change after 12-month treatment; HF—high frequency; HI—Heather index; HR—heart rate; HRV—heart rate variability; LF—low frequency; n.u.—normalized units; pNN50—the proportion of pairs of successive NN intervals that differ by more than 50 ms; rMSSD—the square root of the mean of the sum of the squares of differences between adjacent NN intervals; SDNN—standard deviation of the average of NN intervals; SI—stroke index; SVRI—systemic vascular resistance index; TAC—total arterial compliance; TP—total power; VI—velocity index.

## Data Availability

Data are available upon request.

## Supplementary Materials

The following supporting information can be downloaded at: https://www.mdpi.com/article/10.3390/jcm11133767/s1, Figure S1. Treatments used during the study period: Beta-blockers, angiotensin-converting enzyme inhibitors (ACE-I), angiotensin receptor blockers (ARB), diuretics, calcium channel blockers (CCB). Figure S2. The effects of treatment on the heart rate (HR), systolic blood pressure (SBP), and diastolic blood pressure (DBP) (*p*-values for significant changes). Table S1. Echocardiographic variables. Table S2. Heart rate variability parameters.
