# Supplementary material for: Associations between Heart Rate Variability Parameters and Hemodynamic Profiles in Patients with Primary Arterial Hypertension, Including Antihypertensive Treatment Effects
Source: J Clin Med. 2022 Jun 29;11(13):3767. doi: 10.3390/jcm11133767 (PMC9267277; doi:10.3390/jcm11133767)
Supplement: Supplementary file 1 [file jcm-11-03767-s001.zip › jcm-1770119-supplementary.pdf]

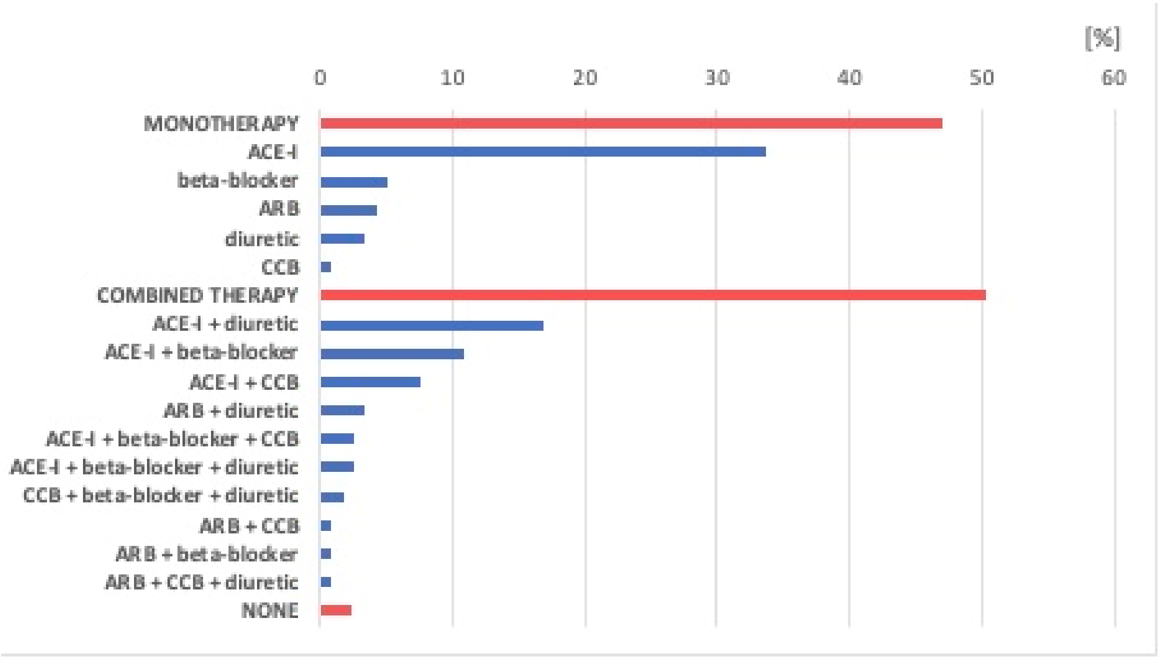

Figure S1. Treatments used during the study period*:* Beta-blockers, angiotensin converting enzyme inhibitors (ACE-I), angiotensin receptor blockers (ARB), diuretics, calcium channel blockers (CCB).

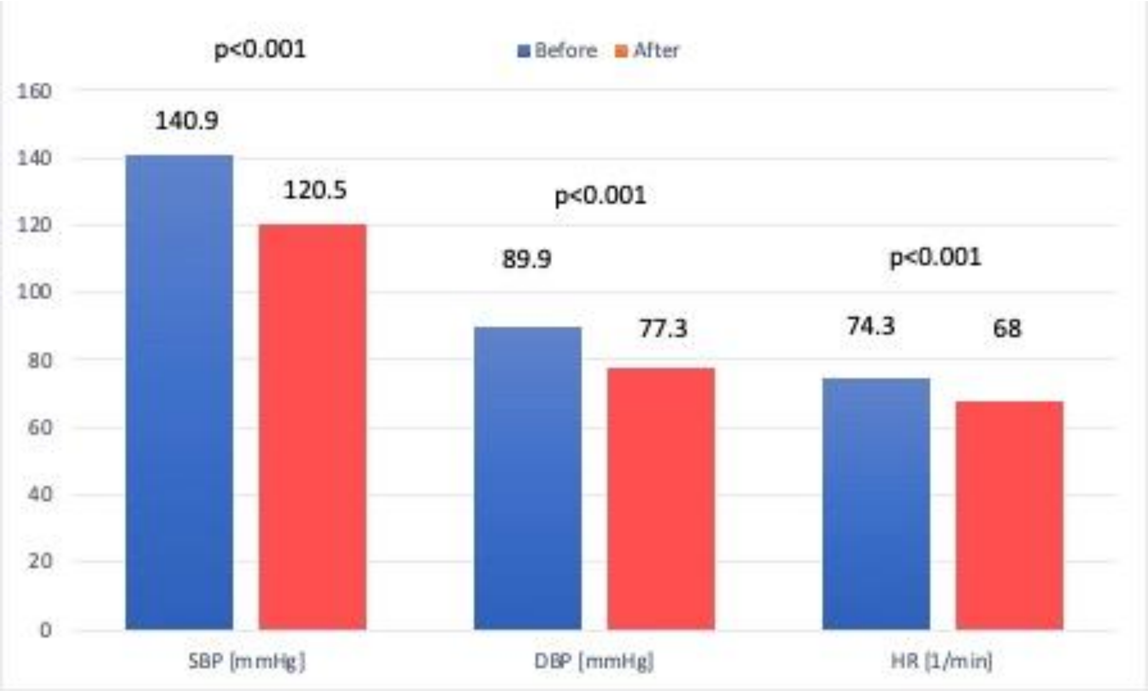

Figure S2. The effects of treatment on the heart rate (HR), systolic blood pressure (SBP), and diastolic blood pressure (DBP) (p-values for significant changes)

Table S1. Echocardiographic variables

Echocardiographic variable	mean ± SD / n (%)
RVEDd [mm]	28.6 ± 3.3
IVSDd [mm]	10.0 ± 1.1
LVEDd [mm]	48.7 ± 3.6
PWDd [mm]	10.0 ±1.2
IVSSd [mm]	13.4 ± 1.4
LVESd [mm]	31.3 ± 2.5
PWSd [mm]	14.5 ± 1.6
LA [mm]	36.9 ± 3.2
AoA [mm]	31.3 ± 3.4
LVMI [g/m^2]	88.9 ± 15.3
LVEF [%]	65.3 ± 3.1
E/A [-]	1.1 ± 0.3
e' [cm/s]	9.7 ± 2.6
E/e' [-]	7.2 ± 1.7
GLS [%]	-18.1 ± 2.8

AoA – diameter of the ascending aorta; e' – mitral septal annulus early diastolic velocity; E/A – the ratio of the early (E) and late (A) mitral flow; E/e' – the ratio of early mitral flow (E) and mitral septal annulus early diastolic velocity (e'); GLS – global longitudinal strain; IVSDd – interventricular septum diastolic diameter; IVSSd – interventricular septum systolic diameter; LA – diameter of the left atrium; LVEDd – left ventricular end-diastolic diameter; LVEF – left ventricular ejection fraction; LVESD – left ventricular end-systolic diameter; LVMI – left ventricular mass index; PWDd – posterior wall end-diastolic diameter; PWSd - posterior wall systolic diameter; RVEDd – right ventricular end-diastolic diameter; SD – standard deviation;

Table S2. Heart rate variability parameters

HRV parameter	mean ± SD
SDNN_24h [ms]	140.8 ± 36.6
SDNN_day [ms]	114.4 ± 30.0
SDNN_night [ms]	92.6 ± 28.4
rMSSD_24h [ms]	34.6 ± 14.3
rMSSD_day [ms]	29.7 ± 11.7
rMSSD_night [ms]	44.4 ± 22.0
pNN50_24h [%]	8.6 ± 8.0
pNN50_day [%]	5.9 ± 6.3
pNN50_night [%]	15.8 ± 14.5
LF/HF_day [-]	4.1 ± 3.2
LF/HF_night [-]	2.5 ± 2.3
LF_day [n.u.]	70.5 ± 13.34
LF_night [n.u.]	58.5 ± 18.0
HF_day [n.u.]	23.5 ± 10.9
HF_night [n.u.]	35.3 ± 16.8
TP_day [ms^2]	2908.0 ± 2258.0
TP_night [ms^2]	3025.2 ± 2664.5

HF – high frequency; HRV – heart rate variability; LF – low frequency; n.u. – normalized units; pNN50 – the proportion of pairs of successive NN intervals that differ by more than 50 ms; rMSSD – the square root of the mean of the sum of the squares of differences between adjacent NN intervals; SD – standard deviation; SDNN – standard deviation of the average of NN intervals; TP – total power;

Table S3. Correlations of heart rate variability parameters with systolic and diastolic blood pressure for baseline absolute values and changes (delta) after 12-month treatment.

Baseline - R (correlation coefficient)		
HRV parameters	SBP [mmHg]	DBP [mmHg]
SDNN_24 h [ms]	-0.06	-0.23**
SDNN_day [ms]	-0.02	-0.21*
SDNN_night [ms]	-0.03	-0.21*
rMSSD_24 h [ms]	-0.07	-0.23**
rMSSD_day [ms]	-0.05	-0.21*
rMSSD_night [ms]	-0.03	-0.19*
pNN50_24h [%]	-0.06	-0.21*
pNN50_day [%]	-0.05	-0.17*
pNN50_night [%]	-0.06	-0.22*
LF/HF_day [-]	-0.04	0.01
LF/HF_night [-]	0.08	0.11
LF_day [n.u.]	-0.05	0.03
LF_night [n.u.]	0.09	0.13
HF_day [n.u.]	0.04	0.01
HF_night [n.u.]	-0.08	-0.07
TP_day [ms^2]	-012	-0.13
TP_night [ms^2]	-0.01	-0.09
Change after treatment (delta) - R (correlation coefficient)		
	SBP [mmHg]	DBP [mmHg]
SDNN_24h [ms]	-0.28**	-0.31#
SDNN_day [ms]	-0.25**	-0.36#
SDNN_night [ms]	-0.11	-0.34
rMSSD_24 h [ms]	-0.21*	-0.40#
rMSSD_day [ms]	-0.19	-0.42#
rMSSD_night [ms]	-0.16	-0.31#
pNN50_24h [%]	-0.04	-0.10
pNN50_day [%]	-0.01	-0.08
pNN50_night [%]	-0.06	-0.10
LF/HF_day [-]	-0.05	-0.01
LF/HF_night [-]	-0.10	-0.03
LF_day [n.u.]	0.03	-0.02
LF_night [n.u.]	-0.04	-0.12
HF_day [n.u.]	0.11	0.10
HF_night [n.u.]	0.15	0.14
TP_day [ms^2]	-0.17	-0.14
TP_night [ms^2]	0.02	-0.13

Statistically significant correlations: *p < 0.05; ** p < 0.01; # p < 0.001

DBP – diastolic blood pressure; HF – high frequency; HRV – heart rate variability; LF – low frequency; n.u. – normalized units; pNN50 – the proportion of pairs of successive NN intervals that differ by more than 50 ms; rMSSD – the square root of the mean of the sum of the squares of differences between adjacent NN intervals; SDNN – standard deviation of the average of NN intervals; SBP – systolic blood pressure; TP – total power

Table S4. Correlations of heart rate variability parameters with echocardiographic parameters of LV systolic and diastolic function for baseline absolute values and changes (delta) after 12-month treatment.

Baseline - R (correlation coefficient)					
HRV parameters	LVEF	E/A	e’	E/e’	GLS
SDNN_24h [ms]	-0.01	0.36#	0.23**	-0.03	-0.09
SDNN_day [ms]	0.07	0.34#	0.23**	-0.09	-0.11
SDNN_night [ms]	-0.06	0.25**	0.19*	-0.02	-0.06
rMSSD_24 h [ms]	-0.05	0.41#	0.24**	-0.04	-0.01
rMSSD_day [ms]	0.02	0.41#	0.26**	-0.08	-0.08
rMSSD_night [ms]	-0.12	0.37#	0.22*	-0.02	0.03
pNN50_24h [%]	-0.07	0.44#	0.23**	-0.01	-0.01
pNN50_day [%]	0.01	0.45#	0.26**	-0.05	-0.05
pNN50_night [%]	-0.14	0.40#	0.22**	0.01	0.01
LF/HF_day [-]	0.16	-0.01	-0.01	-0.05	-0.03
LF/HF_night [-]	0.03	-0.16	-0.10	-0.01	0.01
LF_day [n.u.]	0.05	0.05	0.04	-0.08	-0.06
LF_night [n.u.]	0.02	-0.12	-0.06	-0.02	0.01
HF_day [n.u.]	-0.17	0.04	0.05	0.05	-0.01
HF_night [n.u.]	-0.04	0.16	0.13	-0.01	-0.01
TP_day [ms^2]	0.06	0.32#	0.26**	-0.17	-0.09
TP_night [ms^2]	-0.17*	0.28**	0.23**	-0.06	0.03
Change after treatment (delta) - R (correlation coefficient)					
HRV parameters	LVEF	E/A	e’	E/e’	GLS
SDNN_24h [ms]	0.14	0.10	0.15	-0.09	0.06
SDNN_day [ms]	0.19*	0.14	0.23*	-0.20*	0.20*
SDNN_night [ms]	0.21*	0.10	-0.04	0.10	0.02
rMSSD_24 h [ms]	0.25**	0.34#	0.13	-0.03	0.19
rMSSD_day [ms]	0.28**	0.36#	0.18	-0.06	0.25*
rMSSD_night [ms]	0.19*	0.32#	0.11	-0.05	0.12
pNN50_24h [%]	0.22*	0.38#	0.18	-0.03	0.16
pNN50_day [%]	0.22*	0.35#	0.19*	-0.05	0.21*
pNN50_night [%]	0.17	0.36	0.11	-0.01	0.06
LF/HF_day [-]	0.16	-0.01	0.02	-0.07	-0.25*
LF/HF_night [-]	0.13	-0.04	0.01	-0.01	-0.16
LF_day [n.u.]	-0.10	0.07	-0.02	0.10	0.13
LF_night [n.u.]	-0.01	-0.15	-0.02	-0.03	-0.09
HF_day [n.u.]	-0.01	-0.17	-0.02	-0.03	-0.12

TP_day [ms^2]	0.01	0.20*	0.06	0.02	0.15
TP_night [ms^2]	0.14	0.17	0.10	-0.06	0.03

Statistically significant correlations: *p < 0.05; ** p < 0.01; # p < 0.001
delta – change after 12-month treatment; e' – mitral septal annulus early diastolic velocity; E/A – the ratio of the early (E) and late (A) mitral flow; E/e' – the ratio of early mitral flow (E) and mitral septal annulus early diastolic velocity (e'); GLS – global longitudinal strain; HF – high frequency; HRV – heart rate variability; LF – low frequency; LVEF – left ventricular ejection fraction; ns – not statistically significant; n.u. – normalized units; pNN50 – percentage of NN50; rMSSD – the square root of the mean of the sum of the squares of differences between adjacent NN intervals; SDNN – standard deviation of the average of NN intervals; TP – total power
